# Nutritional Composition, Volatile Compounds, and Phenolic Constituents of *Ficus mathewsii* and *Ficus benjamina* Infructescences (Moraceae)

**DOI:** 10.3390/molecules31193563

**Published:** 2026-10-07

**Authors:** Josias Martins Dos Anjos Cruz, Débora Nogueira Cavalcante, Renilto Frota Corrêa, Jerusa Araújo Quintão Arantes Faria, Jaime Paiva Lopes Aguiar, Francisca Das Chagas Do Amaral Souza, Edgar Aparecido Sanches, Pedro Henrique Campelo, Jaqueline De Araújo Bezerra

**Affiliations:** 1 Department of Teaching, Research, and Extension (DEPE), Federal Institute of Education, Science and Technology of Amazonas (IFAM), Tefé 69555-265, AM, Brazil; 2 Graduate Program in Chemistry (PPGQ), Federal University of Amazonas (UFAM), Manaus 69077-000, AM, Brazil; 3 Center for Studies in Science and Technology in the Amazon (NECTAM), Manaus 69020-120, AM, Brazil; 4 Graduate Program in Materials Science and Engineering (PPGCEM), Federal University of Amazonas (UFAM), Manaus 69077-000, AM, Brazil; 5 Graduate Program in Basic and Applied Immunology (PPGIBA), Federal University of Amazonas (UFAM), Manaus 69077-000, AM, Brazil; 6 Laboratory of Physical Chemistry of Food (LFQA), National Institute of Amazonian Research (INPA), Manaus 69067-375, AM, Brazil; 7 Department of Materials Engineering (DEMa), Federal University of São Carlos (UFSCar), São Carlos 13565-905, SP, Brazil; 8 Department of Food Technology (DTA), Federal University of Viçosa (UFV), Viçosa 36570-900, MG, Brazil; 9 Coordination of Technology and Innovation (COTEI), National Institute of Amazonian Research (INPA), Manaus 69067-375, AM, Brazil

**Keywords:** Amazonian fruits, antioxidant capacity, cell viability, figs, NMR, phenolic compounds, UPLC–QTOF–MS/MS

## Abstract

*Ficus* species are recognized for their nutritional and phytochemical diversity; however, the infructescences of *Ficus benjamina* and *Ficus mathewsii* remain underutilized. This study aimed to evaluate the nutritional composition, volatile and phenolic profiles, radical-scavenging capacity, and effects on cell viability of these two species. Both species exhibited distinct nutritional profiles: *F. benjamina* showed higher carbohydrate, energy, and mineral contents, whereas *F. mathewsii* exhibited 3.3-fold higher DPPH^•^ radical-scavenging capacity and 4.3-fold higher total phenolic content than *F. benjamina*. The volatile profiles shared several compounds, with only a few detected exclusively in one species. Sesquiterpenes exhibited the greatest diversity, with 16 compounds, accounting for more than 47% of the total chromatographic area in both species. In the cell viability assay, only *F. benjamina* at 50% caused a reduction in cell viability to approximately 50%. Ten phenolic metabolites were annotated by UPLC–QTOF–MS/MS, including six flavonols (quercetin, kaempferol, rutin, narcissin, nicotiflorin, and quercitrin), two flavones (rhoifolin and schaftoside), one flavanone (naringin), and one phenolic acid (chlorogenic acid). The presence of kaempferol and chlorogenic acid was corroborated by 1D and 2D NMR analyses. Overall, *F. mathewsii* exhibited higher phenolic content and radical-scavenging capacity, whereas *F. benjamina* showed higher mineral content. Further studies on nutrient bioavailability are needed to assess the potential applications of these infructescences in the food, nutraceutical, or pharmaceutical sectors.

## 1. Introduction

The genus *Ficus* is the largest genus in the family Moraceae, comprising more than 1000 species distributed primarily throughout tropical and subtropical regions [1,2]. Some species are among the earliest plants cultivated by humans, underscoring their historical and cultural significance [3]. The fruits of *Ficus*, commonly known as figs, are syconia-type infructescences consisting of a fleshy, hollow receptacle containing numerous flowers within its internal cavity. These infructescences feature a thin, soft skin and a fleshy edible tissue, with pollination in several species being mediated by highly specialized wasps [2].

In addition to their dietary value, various *Ficus* species are used in traditional medicine to treat conditions such as anemia, kidney stones, diarrhea, fever, infections, and inflammation [4,5,6]. This ethnopharmacological relevance is supported by extensive scientific literature reporting a wide range of biological properties. In vitro assays have demonstrated radical-scavenging, antimicrobial, anti-inflammatory, immunomodulatory, and anticancer activities. Furthermore, in vivo studies have reported antioxidant, anti-inflammatory, antidiabetic, anti-obesity, and neuroprotective effects, including effects relevant to Alzheimer’s disease [7,8], although clinical evidence remains lacking.

This pharmacological versatility stems from a complex chemical composition. The infructescences of species within this genus contain a wide variety of metabolites, including fatty acids, organic acids, sugars, alkaloids, amines, carotenoids, steroids, glycosides, proteins, saponins, triterpenes, monoterpenes, sesquiterpenes, tocopherols, vitamins, and, particularly, phenolic compounds [7,9,10,11]. Among these constituents, phenolic acids and flavonoids are among the major classes of phenolic compounds reported in the genus *Ficus* [7]. These metabolites have attracted considerable interest because of their ability to participate in processes associated with oxidative stress and various cellular mechanisms, contributing to the biological and nutraceutical potential attributed to species of this genus [12,13,14].

Among *Ficus* species, *Ficus carica* is the best known and most widely cultivated, which is particularly valued for its sweet and succulent infructescences, which are consumed in many regions of the world [15]. Currently, a variety of products derived from *F. carica* are commercially available, including fresh and dried fruits, jams, fruit preserves, canned fruits, candied fruits, concentrates, powders, and ingredients used in bakery and confectionery products. In addition, the genus *Ficus* has shown promise for the development of various patented products, including food technologies such as calcium-supplemented animal feed, functional teas, jams, and wine, as well as pharmaceutical patents focusing on tumor cell inhibition and sunscreen formulations [7]. The existence of this range of products and patented technologies provides a reference for exploring new applications for species that remain commercially underexplored and poorly characterized in terms of their chemical composition and biological properties, such as *Ficus benjamina* and *Ficus mathewsii*.

*Ficus benjamina* L. is an evergreen tree native to South Asia and northern Australia, widely cultivated for urban greening in Brazil, where it is commonly known as figueira-benjamim [16]. Chemical studies on this species are scarce and predominantly focused on its leaves, including the evaluation of in vitro antioxidant and antimicrobial activities [17], its application in nanoparticle synthesis [18], its antimalarial potential attributed to ursolic acid and lupeol [19] and the profiling of its oxygenated volatile organic compounds [20]. In contrast, *Ficus mathewsii* (Miq.) Miq. is native to South America [1], but even its leaves remain unexplored.

To the best of our knowledge, the only literature report on the chemistry of the infructescences of these two species is a preliminary study that highlights their radical-scavenging capacity and reveals the presence of α-glucose, β-glucose, fructose, as well as citric, malic, quinic, shikimic, and hydroxybenzoic acids in their aqueous extracts [21].

In this context, the infructescences of *F. benjamina* and *F. mathewsii* were selected for comparative evaluation because both species occur in the Brazilian Amazon, belong to the same genus, produce substantial amounts of infructescences that are frequently discarded as urban waste, and remain poorly characterized in terms of their chemical composition and bioactive potential. Therefore, this study aimed to characterize the physicochemical and phytochemical profiles of the infructescences of *F. benjamina* and *F. mathewsii* and to evaluate their free-radical-scavenging capacity and effects on cell viability. It should be emphasized that the study was designed as a comparative investigation and was not intended to assess combined, synergistic, or complementary effects between the two species. Thus, the findings may support future strategies for the valorization of these infructescences.

## 2. Results

### 2.1. Proximate Composition

The proximate composition and energy values of fresh *F. benjamina* and *F. mathewsii* infructescences are summarized in Table 1. As expected for fresh botanical matrices, water constituted the major fraction in both species, with moisture levels being significantly higher in *F. mathewsii* (76.09 ± 0.99 g/100 g) than in *F. benjamina* (66.30 ± 1.08 g/100 g). Carbohydrates represented the primary macronutrient, showing significantly higher concentrations in *F. benjamina*. Protein (1.08 ± 0.04 vs. 0.94 ± 0.04 g/100 g) and lipid contents (0.67 ± 0.03 vs. 0.47 ± 0.02 g/100 g) were low in both species. Additionally, *F. benjamina* exhibited a significantly higher ash content (1.56 ± 0.05 g/100 g) compared to *F. mathewsii* (0.99 ± 0.02 g/100 g). The calculated energy values were also significantly different between the species.

### 2.2. Mineral Composition

The mineral composition of the freeze-dried infructescences is shown in Table 2. Mg, K, and Ca were the predominant macrominerals detected in both species. Mg was the most abundant mineral, with concentrations of 14.31 ± 0.25 g kg^−1^ in *F. benjamina* and 12.05 ± 0.04 g kg^−1^ in *F. mathewsii*. Among the trace elements, Zn showed the highest concentrations, followed by Mn and Fe. *F. benjamina* exhibited significantly higher concentrations of Mg, Ca, Mn, Zn, and Fe than *F. mathewsii*. K was the only mineral that did not differ significantly between the species. P and Cu were detected but were not quantifiable under the analytical conditions employed.

### 2.3. GC–MS Analysis of Volatile Compounds

The yield of the volatile fraction obtained from the infructescences via hydrodistillation was below the limit of direct gravimetric determination, with no visible phase observed after distillation. Consequently, the volatile compounds were recovered by in-system partitioning with hexane. Given the residual nature of the isolated fraction and the impracticality of quantifying the initial extract mass, the GC–MS data for the volatile compounds identified in the infructescences are presented in Table 3 as relative abundance (% peak area), describing the qualitative phytochemical profile and the relative distribution of the identified constituents. GC–MS analysis resulted in the identification of 23 volatile compounds, comprising 19 in *F. benjamina* and 20 in *F. mathewsii*, accounting for 70.62% and 76.22% of the total chromatographic area, respectively.

Sesquiterpenes represented the largest relative peak-area class in both species, accounting for 52.80% of the total chromatographic area in *F. benjamina* and 47.66% in *F. mathewsii*. In *F. benjamina*, the compounds with the highest relative peak areas were *β*-selinene (7.34%), *δ*-cadinene (5.92%), and *cis*-α-bergamotene (5.42%). In *F. mathewsii*, *β*-elemene (7.56%), *β*-farnesene (5.83%), and *α*-selinene (5.83%) showed the highest relative peak areas.

Sesquiterpenoids represented 12.78% and 17.65% of the chromatographic area in *F. benjamina* and *F. mathewsii*, respectively. *γ*-eudesmol, α-eudesmol, and neointermedeol were detected in both species, whereas (*E*)-isovalencenol was detected only in *F. mathewsii*. Esters accounted for 5.04% in *F. benjamina* and 10.91% in *F. mathewsii*. Methyl palmitate, methyl linoleate, and ethyl linoleate were detected among this class.

### 2.4. Cell Viability Assay

**Figure 1:**
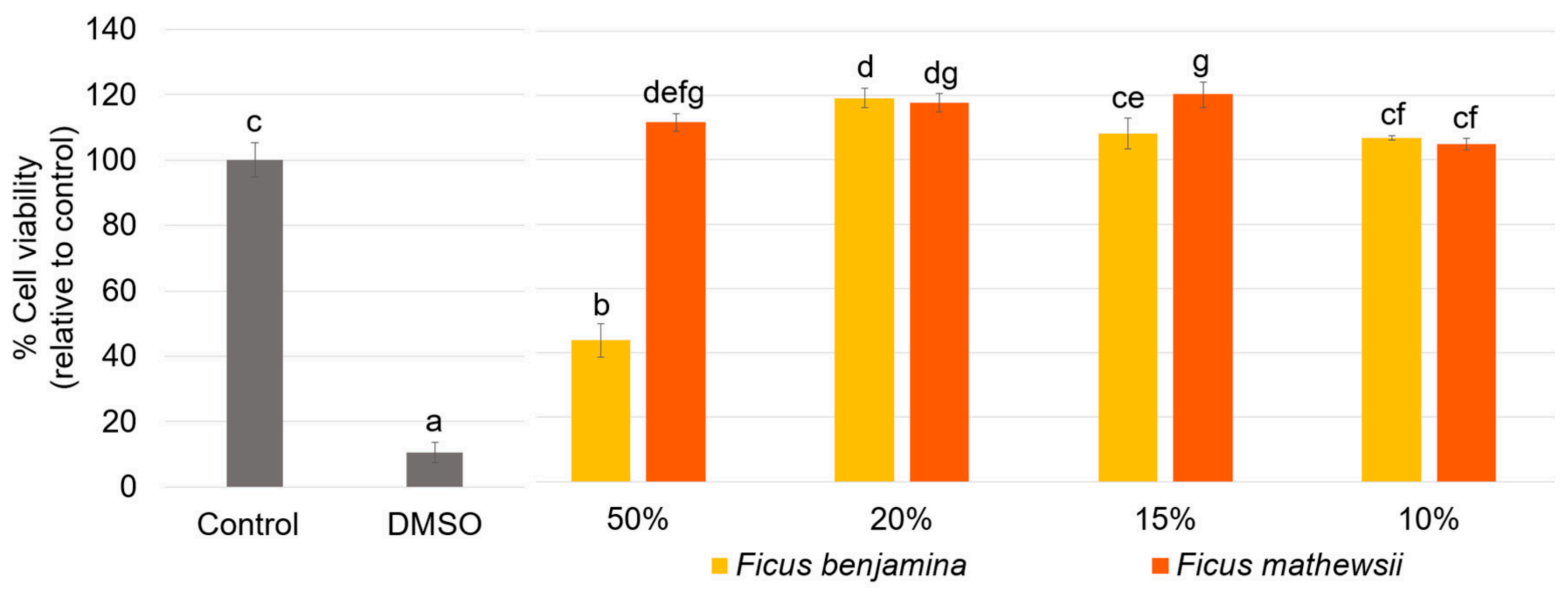
Cell viability of BEAS-2B cells in response to *Ficus benjamina* and *Ficus mathewsii* infructescences. Different letters indicate significant differences among means according to Tukey’s test (*p* < 0.05).

Cell viability of non-tumorigenic human bronchial epithelial cells (BEAS-2B cells) following exposure to the infructescences of *F. benjamina* and *F. mathewsii* is shown in Figure 1. The samples were evaluated at concentrations corresponding to 50, 20, 15, and 10% of the fresh infructescence. At the highest concentration evaluated (50%), *F. benjamina* produced a reduction in cell viability to approximately 50% relative to the untreated control. At the other concentrations, no significant reduction in cell viability was observed for *F. benjamina*. Similarly, *F. mathewsii* did not significantly reduce cell viability at the concentrations evaluated. An increase in cell viability relative to the control was observed at some concentrations.

### 2.5. Radical-Scavenging Capacity, Total Phenolic Content, and Carotenoids

The scavenging capacity for DPPH^•^ and ABTS^•+^ radicals, total phenolic content (TPC), and total carotenoid content (TCC) of the hydromethanolic extracts are presented in Table 4. Significant differences were observed between the two species in both radical-scavenging assays and in TPC. The antioxidant capacity was significantly higher in *F. mathewsii*, which exhibited DPPH^•^ and ABTS^•+^ scavenging activities of 998.3 ± 4.6 and 1572.9 ± 21.0 μmol TE/g, respectively. In comparison, *F. benjamina* yielded lower values of 300.5 ± 2.1 μmol TE/g for DPPH^•^ and 1152.4 ± 18.9 μmol TE/g for ABTS^•+^. The TPC was also significantly higher in *F. mathewsii* than in *F. benjamina*. No carotenoids were detected in either extract using the analytical method employed.

### 2.6. Extraction Yields and NMR and UPLC–QTOF–MS/MS

The extraction yields obtained with hexane from the freeze-dried samples were 0.72 ± 0.01% for *F. benjamina* and 1.28 ± 0.03% for *F. mathewsii*. Hydromethanolic extraction yielded 3.11 ± 0.01% and 12.47 ± 0.03%, respectively. After solid-phase extraction (SPE), the phenolic fractions yielded 2.5 ± 0.4% for *F. benjamina* and 4.3 ± 0.7% for *F. mathewsii*.

**Figure 2:**
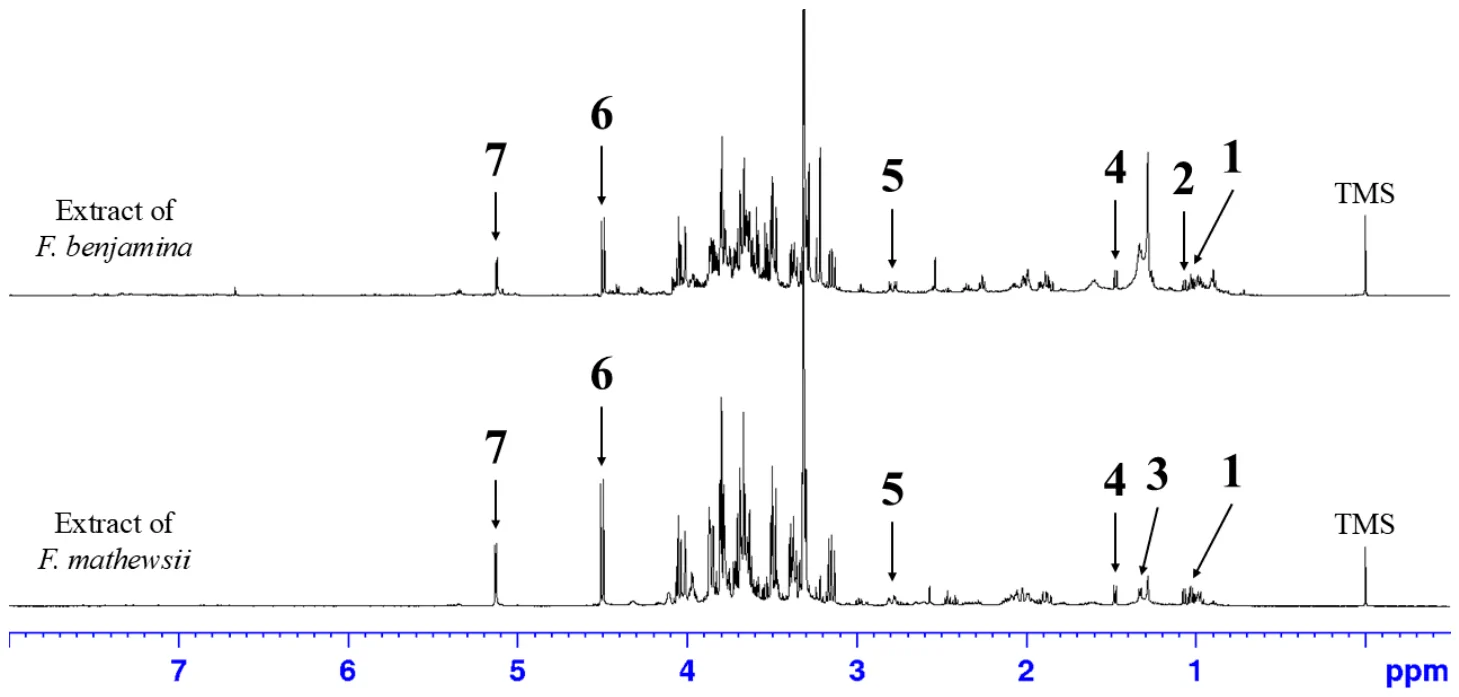
^1^H NMR spectra (500 MHz, CD_3_OD, 0–8 ppm) of hydromethanolic extracts from *F. benjamina* and *F. mathewsii* infructescences.

The ^1^H NMR spectra of the crude extracts showed signals attributable to primary metabolites commonly reported in *Ficus* (Figure 2), including isoleucine (1.02 ppm, d, *J* = 6.9 Hz) (**1**), valine (1.06 ppm, d, *J* = 7.0 Hz; 3.59 ppm, d, *J* = 4.9 Hz; 0.98 ppm, d, *J* = 6.5 Hz) (**2**), threonine (1.32 ppm, d, *J* = 6.4 Hz) (**3**), alanine (1.47 ppm, d, *J* = 7.2 Hz) (**4**), malic acid (2.78 ppm, dd, *J* = 15.9 and 5.3 Hz) (**5**), *β*-glucose (4.50 ppm, d, *J* = 7.8 Hz) (**6**) and α-glucose (5.12 ppm, d, *J* = 3.6 Hz) (**7**) [23,24]. The high abundance of these primary metabolites was associated with substantial signal overlap in the crude extracts. Therefore, SPE was employed prior to the detailed characterization of phenolic constituents.

Qualitative profiling of phenolic compounds was performed using UPLC–QTOF–MS/MS in negative electrospray ionization mode (ESI^−^), allowing the annotation of ten phenolic metabolites (Table 5). In accordance with the Metabolomics Standards Initiative (MSI), all identified constituents were classified as putatively annotated compounds (MSI Level 2), as structural assignment was based strictly on accurate mass measurements and MS/MS fragmentation patterns, without direct comparison to authentic reference standards. The annotated compounds included narcissin, rutin, nicotiflorin, naringin, rhoifolin, schaftoside, quercitrin, chlorogenic acid, quercetin, and kaempferol.

Although no purification steps were performed, NMR analysis of the phenolic fractions provided additional structural evidence for two phenolic compounds annotated by UPLC–QTOF–MS/MS. The characteristic ^1^H NMR signals and corresponding HSQC and HMBC correlations were sufficiently resolved and consistent with literature data for kaempferol [25] and chlorogenic acid [26] (Table 6).

**Table 5:** Spectrometric data obtained by UPLC–QTOF–MS/MS in ESI(-) mode for phenolic compounds annotated in *Ficus benjamina* (Fb) and *Ficus mathewsii* (Fm) based on their fragmentation profiles.

No.	Observed Ion [M-H]- (*m*/*z*)	Mass		Molecular Formula	Mass Error (ppm)	Fragments MS/MS (*m*/*z*)	Compound	Samples		Reference
		Calculated	Theoretical					Fb	Fm	
8	623.1629	624.1707	624.1690	C_28_H_32_O_16_	−2.7	315.0521, 300.0187	Narcissin (Isorhamnetin-3-*O*-rutinoside)	+	-	[27]
9	609.1477	610.1555	610.1533	C_27_H_30_O_16_	−3.6	463.0871, 301.0338, 300.0283, 151.0042	Rutin (quercetin-3-*O*-rutinoside)	+	+	[28]
10	593.1516	594.1594	594.1584	C_27_H_30_O_15_	−1.6	327.0500, 285.0392, 255.0281, 227.0342	Nicotiflorin (kaempferol-3-*O*-rutinoside)	+	-	[29]
11	579.1734	580.1812	580.1792	C_27_H_32_O_14_	−3.4	151.0030, 271.0595, 417.1583, 181.0514	Naringin	+	+	[30]
12	577.1632	578.1650	578.1635	C_27_H_30_O_14_	+2.6	431.0984, 269.0444	Rhoifolin (apigenin 7-*O*-neohesperidoside)	+	-	[31]
13	563.1389	564.1467	564.1479	C_26_H_28_O_14_	+2.1	503.1169, 473.1092, 443.0989, 383.0785	Schaftoside (apigenin 6-C-glucoside 8-*C*-arabinoside)	-	+	[32]
14	447.0905	448.0983	448.1005	C_21_H_20_O_11_	+4.9	300.0278, 301.0339, 271.0315, 255.0261	Quercitrin (quercetin-3-*O*-rhamnoside)	+	+	[28,30]
15	353.0867	354.0945	354.0951	C_16_H_18_O_9_	+1.7	191.0559, 179.0338, 135.0450	Chlorogenic acid	+	+	[33]
16	301.0355	302.0433	302.0426	C_15_H_10_O_7_	−2.3	273.0415, 257.0447, 121.0305	Quercetin	+	+	[34]
17	285.0412	286.0490	286.0477	C_15_H_10_O_6_	−4.5	257.0451, 229.0499, 151.0025	Kaempferol	+	+	[35]

**Table 6:** Secondary metabolites identified by 1D and 2D NMR spectroscopy in the phenolic fractions of *Ficus benjamina* (PFFb) and *Ficus mathewsii* (PFFm) (^1^H, 500 MHz; ^13^C, 125 MHz; CD_3_OD).

Compound	Position	δ_H_ (Multiplicity, *J* in Hz)		HSQC (δ_C_ in ppm)		HMBC Position of C (δC in ppm)
		Fb	Fm	PFFb	PFFm	
Kaempferol	6	6.24 (d, 2.09)	6.20 (d, 2.00)	98.3	98.5	C-5 (158.2), C-10 (103.7), C-9 (156.2)
	8	6.44 (d, 2.08)	6.39 (d, 2.00)	93.4	93.5	C-7 (164.7), C-9 (156.2)
	2′	8.09 (d, 8.59)	7.99 (d, 8.80)	129.3	130.4	C-2 (159.1), C-3′
	3′	6.90 (d, 8.60)	6.85 (d, 8.82)	114.8	118.7	C-1′ (121.6), C-2′. C-4′ (162.4)
	5′	6.90 (d, 8.60)	6.85 (d, 8.82)	114.8	116.7	C-1′ (121.6), C-6′, C-2′
	6′	8.09 (d, 8.59)	7.99 (d, 8.80)	130.3	130.4	C-2 (159.1), C-5′
Chlorogenic acid	2	2.06 (m) and 1.97 (m)	2.01 (m) and 1.92 (m)	41.7	42.2	C-3. C-6. C-7 (181.0)
	3	5.23 (m)	5.29 (m)	72.6	72.3	–
	4	3.51 (dd, 8.51, 3.03)	3.50 (dd, 8.45. 3.10)	71.6	72.1	C-2. C-6
	5	4.04 (m)	4.05 (m)	70.2	69.0	C-1 (76.5), C-3, C-9′ (170.8)
	6	2.06 (m) and 1.97 (m)	2.01 (m) and 1.92 (m)	42.0	42.1	C-2, C-4, C-9′ (170.8)
	2′	7.04 (s)	7.11 (s)	118.7	118.8	C-1′ (129.3), C-3′ (145.4)
	5′	6.83 (d, 8.99)	6.83 (d, 8.64)	116.1	116.3	C-3′ (145.4), C-4′ (149.5), C-6′
	6′	7.00 (d, 8.98)	7.01 (d, 8.67)	122.7	123.2	C-1′ (129.3), C-5′, C-7′
	7′	7.59 (d, 15.45)	7.55 (d, 15.60)	145.3	144.2	C-6′. C-8′, C-9′
	8′	6.23 (d, 15.45)	6.26 (d, 15.60)	113.5	114.0	C-7′. C-9′ (170.8)

The ^13^C chemical shifts were not determined from conventional ^13^C NMR experiments but were obtained from HSQC and HMBC two-dimensional correlation maps.

## 3. Discussion

### 3.1. Nutritional and Mineral Profiles

Proximate composition analysis revealed distinct nutritional profiles between the two *Ficus* species. Both infructescences exhibited high moisture contents, consistent with fresh botanical matrices and previous reports for the genus [36,37,38]. However, *F. benjamina* exhibited higher carbohydrate and total energy contents, as well as a significantly higher ash content, compared with *F. mathewsii*. The values obtained for both species were higher than those reported for *F. subapiculata* in terms of carbohydrate content [38], whereas protein and lipid contents remained relatively low, similar to those reported for other edible *Ficus* species [37,38,39,40], although lower than those reported for *F. carica* [36] and *F. microcarpa* [41].

The marked variation in ash content warrants attention and is directly related to the elemental profile obtained for both species. Overall, the mineral profiles differed significantly between the two species. Ash represents the total inorganic residue remaining after incineration, and the higher ash content observed in *F. benjamina* (1.56 g/100 g) is consistent with its substantially higher concentrations of individual minerals, specifically Mg, Ca, Mn, Zn, and Fe, compared with *F. mathewsii*.

Interestingly, Mg emerged as the predominant macromineral in both infructescences, exceeding K and Ca. Mg plays a fundamental biochemical role in plants as the central atom of the chlorophyll molecule and as an essential cofactor for numerous metabolic enzymes involved in carbohydrate metabolism and energy transfer [42]. The high Mg concentrations detected in these infructescences may reflect the metabolic demands of actively developing reproductive tissues during infructescence development and maturation.

For comparison, the mineral profile observed in the present study differs from that reported for *F. subapiculata*, another species occurring in the Brazilian Amazon, in which K was the predominant macromineral (13.17 g kg^−1^), followed by Ca (8.50 g kg^−1^) and Mg (1.90 g kg^−1^). A marked difference was also observed in the micromineral profile, with Fe (65.90 mg kg^−1^), Mn (43.69 mg kg^−1^), and Zn (25.75 mg kg^−1^) being the predominant microminerals in *F. subapiculata* [38], whereas the predominant microminerals in the species investigated in the present study were Zn, Mn, and Fe, respectively.

These species-specific patterns may also be influenced by differences in environmental growing conditions. *F. benjamina* is frequently cultivated in urbanized and managed environments, whereas *F. mathewsii* occurs *in natural* Amazonian forest habitats, where soil chemistry and nutrient availability may differ. Thus, differences in mineral profiles may reflect environmental factors in addition to species-specific characteristics [43].

From a nutritional perspective, the considerable levels of Mg, K, and Ca highlight the potential of these underutilized infructescences as sources of essential minerals. However, these total elemental concentrations should be interpreted with caution. Their actual nutritional contribution depends not only on dietary intake and serving size but also, importantly, on mineral bioavailability [44]. In plant matrices, inorganic ions may interact with antinutritional factors or become associated with dietary fiber and other components of the food matrix, potentially limiting their bioaccessibility and subsequent intestinal absorption in vivo [45].

### 3.2. Volatile Chemical Profiles

The GC–MS results revealed differences in the qualitative volatile profiles and relative distribution of the detected compounds between the two species, despite the high relative representation of sesquiterpenes in both. In plant reproductive structures, volatile sesquiterpenes play crucial ecological roles, acting as chemical cues for host-specific pollinating wasps [46] and, alongside other volatile compounds, mediating defenses against frugivorous pests and attracting seed dispersers [47,48].

The relative distribution of sesquiterpenes in *F. benjamina* included a higher representation of compounds with bicyclic and tricyclic skeletons, notably *β*-selinene, *δ*-cadinene, and *cis-α*-bergamotene. In contrast, *F. mathewsii* exhibited a distinct terpene profile, with β-elemene, β-farnesene, and α-selinene showing the highest relative peak areas, together with higher relative peak-area contributions from oxygenated sesquiterpenoids (17.65%) and aliphatic esters (10.91%). The detection of (*E*)-isovalencenol exclusively in *F. mathewsii* further illustrates the qualitative differences between the two species.

The high relative representation of sesquiterpenes agrees with the volatile profile previously reported for *F. subapiculata* [38], suggesting that terpenoid metabolites represent an important component of the volatile fraction of *Ficus* infructescences. Nevertheless, factors such as geographic origin, environmental conditions, developmental stage, and other biological variables can significantly influence volatile emission profiles [49].

### 3.3. Cell Viability Assay and Preliminary Safety Assessment

The cell viability assay indicated limited effects of the tested samples on BEAS-2B cells under the experimental conditions. The exception was *F. benjamina* at the highest evaluated concentration (50% fresh infructescences equivalent), which reduced cell viability to approximately 50% relative to the untreated control.

The methodological choice to evaluate cell viability using a reconstituted suspension of the infructescences matrix, rather than extracts isolated with organic solvents, was intended to reflect the overall, complex composition of the whole plant material. This approach is particularly relevant in preliminary safety evaluations of infructescences intended for fresh (*in natura*) consumption. However, this strategy carries crucial methodological implications. While organic extracts concentrate and solubilize specific phytochemical classes, direct cell exposure to an in vitro plant suspension depends strictly on aqueous solubility and the spontaneous release of constituents into the culture medium. In vivo, oral consumption involves gastrointestinal digestion, enzymatic transformation, and selective intestinal absorption, all of which profoundly alter the bioavailability and metabolic form of phytochemicals. Under the applied in vitro conditions, poorly soluble or strongly matrix-bound lipophilic metabolites may not have been sufficiently released, thereby limiting their direct interaction with epithelial cells. Consequently, the absence of a detectable reduction in cell viability for *F. mathewsii* (as well as for lower concentrations of *F. benjamina*) should be interpreted with caution, as it may reflect the restricted release of compounds into the culture medium rather than an absolute absence of bioactivity or toxicity.

The reduction in cell viability observed for *F. benjamina* at the highest tested concentration indicates a cellular response under the experimental conditions used. This response may be associated with differences in the overall chemical composition of the two species. *F. mathewsii* was characterized by higher phenolic content and greater radical-scavenging capacity, whereas *F. benjamina* exhibited higher mineral ash content, as well as characteristic volatile sesquiterpenes, such as β-selinene and δ-cadinene, and glycosylated flavonoids, including narcissin and nicotiflorin.

### 3.4. Radical-Scavenging Capacity and Phenolic Content

*F. mathewsii* exhibited substantially greater radical-scavenging capacity than *F. benjamina*, showing a 3.3-fold higher DPPH^•^ scavenging activity (998.3 vs. 300.5 µmol TE/g) and a higher ABTS^•+^ scavenging capacity (1572.9 vs. 1152.4 µmol TE/g). This difference was accompanied by an approximately 4.3-fold higher total phenolic content (TPC) in *F. mathewsii* (659.1 mg GAE/g) than in *F. benjamina* (152.7 mg GAE/g), suggesting that phenolic constituents may contribute to the greater radical-scavenging capacity observed in *F. mathewsii*. These findings highlight this species as a particularly promising matrix for further investigation of its phenolic composition and bioactive potential.

However, the association between TPC and radical-scavenging capacity should not be interpreted as a direct causal relationship. Plant extracts are complex mixtures of metabolites that may act individually or through additive and synergistic interactions. Moreover, the Folin–Ciocalteu assay is not specific to phenolic compounds, as other reducing substances may also contribute to the analytical response [50]. Therefore, TPC values should be regarded as an estimate of compounds reactive toward the Folin–Ciocalteu reagent rather than as an absolute measure of total phenolic content. Likewise, DPPH^•^ and ABTS^•+^ assays assess radical-scavenging capacity under specific in vitro conditions and should not be interpreted as direct measures of in vivo antioxidant activity [51].

The differences observed between the DPPH^•^ and ABTS^•+^ assays may also be related to the distinct chemical characteristics of these methods. The DPPH^•^ assay is particularly responsive to the ability of antioxidants to transfer hydrogen atoms and/or electrons to the DPPH^•^ radical and is influenced by the polarity of the reaction medium and the accessibility of the compounds to the radical. In contrast, the ABTS^•+^ assay primarily involves electron-transfer processes, although hydrogen-atom transfer mechanisms may also contribute to the reaction. Because ABTS^•+^ can react with antioxidant compounds across media of different polarities, this assay may respond to a broader range of reducing species, including both phenolic and non-phenolic compounds [52].

The greater radical-scavenging capacity of *F. mathewsii* may be related, at least in part, to its phenolic composition, as characterized by UPLC–QTOF–MS/MS and NMR. Among the annotated metabolites, quercetin, rutin, kaempferol, and chlorogenic acid possess structural features associated with antioxidant activity. In particular, the presence of a 3′,4′-dihydroxylated (catechol) moiety in the B-ring of flavonoids such as quercetin and rutin may favor hydrogen-atom donation and resonance stabilization of the resulting phenoxyl radicals, thereby contributing to their radical-scavenging capacity [53,54].

Although the assays employed here assess the chemical reactivity of plant extracts toward synthetic radicals under defined experimental conditions and do not allow direct extrapolation to physiological in vivo antioxidant status, the results obtained, together with the characterized phenolic profile, indicate that *F. mathewsii* represents a relevant source of phenolic metabolites and warrants further investigation of its chemical composition and the bioactive potential of their individual constituents.

### 3.5. Phenolic Metabolites Revealed by UPLC–QTOF–MS/MS and NMR

The combination of SPE, UPLC–QTOF–MS/MS, and NMR was particularly useful for characterizing the phenolic fraction of the two *Ficus* infructescences. The ^1^H NMR spectra of the crude extracts showed intense signals attributable to primary metabolites, particularly amino acids and sugars. Their abundance can complicate the detection and interpretation of signals from minor secondary metabolites. The use of SPE therefore provided a practical strategy for reducing matrix interference and facilitating the characterization of phenolic constituents.

The UPLC–QTOF–MS/MS data revealed a chemically diverse phenolic profile comprising flavonoid glycosides, flavonoid aglycones, and phenolic acids. The annotation of rutin at *m*/*z* 609.1477 was supported by the presence of characteristic product ions, including *m*/*z* 463.0871, 301.0338, 300.0283, and 151.0042. Similarly, the ion at *m*/*z* 353.0867 was annotated as chlorogenic acid, with fragments at *m*/*z* 191.0559, 179.0338, and 135.0450. Quercetin and kaempferol were observed at *m*/*z* 301.0355 and 285.0412, respectively. The detection of flavonoid glycosides such as rutin, quercitrin, narcissin, nicotiflorin, and rhoifolin indicates considerable structural diversity among the phenolic metabolites.

Although UPLC–QTOF–MS/MS analysis revealed the presence of 10 metabolites in the SPE fraction, only two of them, kaempferol and chlorogenic acid, exhibited NMR signals that were sufficiently intense and well resolved to allow their structural assignment. This finding is directly related to the lower sensitivity of NMR compared with mass spectrometric techniques, as the observation and unambiguous assignment of NMR signals depend, among other factors, on the concentration of the metabolites in the sample. Thus, although UPLC–QTOF–MS/MS analysis revealed a broader diversity of phenolic metabolites, the detection and assignment of kaempferol and chlorogenic acid by NMR suggest that these compounds are among the most abundant phenolic constituents of the SPE fraction [55].

### 3.6. Limitations and Future Research

Several limitations should be considered when interpreting the present findings. First, the study was based on samples collected from different geographic locations, and therefore the relative contributions of species-specific and environmental factors to the observed differences could not be fully separated. Controlled sampling across different geographic locations, seasons, and developmental stages would be useful to better assess the extent to which environmental and biological factors contribute to the observed chemical and biological differences.

Second, the GC–MS results represent relative chromatographic abundances based on integrated peak areas and should not be interpreted as absolute concentrations of individual volatile compounds. Likewise, the phenolic metabolites identified by UPLC–QTOF–MS/MS were annotated based on their analytical characteristics, and only kaempferol and chlorogenic acid received additional structural support from NMR analyses. Individual metabolites were not quantitatively determined in the present study. Future investigations should therefore include quantitative determination of the individual metabolites to provide a more comprehensive assessment of the chemical composition of these infructescences.

The cell viability assay also provides a preliminary assessment under in vitro conditions and does not establish in vivo safety, toxicological effects, or the mechanisms underlying the observed cellular responses. Although a reduction in cell viability was observed for *F. benjamina* at the highest concentration tested, the present study does not establish a dose–response relationship or allow the contribution of specific compounds to the observed cellular response to be determined. The volatile and phenolic constituents were not individually evaluated for their biological effects. Further studies using characterized extracts, isolated compounds, additional cellular models, and complementary toxicity endpoints are therefore needed to clarify the mechanisms underlying the observed responses and their potential toxicological relevance.

Additionally, the slight increases in metabolic activity observed at certain lower concentrations should not be interpreted as evidence of cell proliferation, because the resazurin assay primarily reflects cellular metabolic activity and viability rather than directly measuring cell proliferation. Complementary assays would therefore be necessary to determine whether such changes are associated with alterations in cell number or other cellular processes.

Finally, further studies should address the bioaccessibility and bioavailability of the identified compounds and minerals, as well as their biological effects using complementary experimental models and endpoints. Such investigations, together with controlled sampling across different locations and developmental stages and mechanistic studies, would help clarify the factors underlying the chemical and biological differences observed between the two *Ficus* species and better define the potential applications of their infructescences.

## 4. Materials and Methods

### 4.1. Sample Collection and Preparation

The infructescences of *Ficus benjamina* (Fb) were collected in Manaus, Amazonas, Brazil (3.1339165° S, 60.0127219° W), while those of *Ficus mathewsii* (Fm) were collected in a rural area of Iranduba, Amazonas, Brazil (3.0951107° S, 60.3741374° W). The plant materials were collected under SisGen authorization AE61B3B. Taxonomic identification was performed by Drs. Valdely Ferreira Kinupp and Renilto Frota Corrêa; however, voucher specimens were not deposited.

The collected infructescences were selected and washed under running tap water. Approximately 30 g of fresh samples were separated for proximate composition analyses. The remaining plant material was freeze-dried at 233.15 K and −93.33 kPa for 72 h using an SL-404 freeze-dryer (Solab, Piracicaba, SP, Brazil) to obtain the dried material, which was subsequently ground and used for the cell viability assay, mineral composition analysis, and extract preparation.

### 4.2. Proximate Composition Analysis

The infructescences were analyzed for moisture content (oven drying at 105 °C), ash content (incineration at 550.0 °C), protein content (classical Kjeldahl method), and lipid content (Soxhlet extraction with hexane for 8 h) [56]. Carbohydrate content was determined by difference [43], and the total energy value was calculated using Equation (1) [36].

(1) Energy(kcal)=4×(gprotein+gcarbohydrate)+9×(glipid)

### 4.3. Mineral Composition Analysis

Mineral quantification was carried out according to the procedures established by the Adolfo Lutz Institute [56]. Briefly, lyophilized samples (0.5 g) were digested with 6 mL of concentrated HNO_3_ using a microwave digestion system (MARS–Xpress; CEM Corporation, Matthews, NC, USA). Following digestion, the samples were cooled to room temperature and diluted with deionized water. The concentrations of the mineral elements were determined by direct measurement using an atomic absorption spectrophotometer (SpectrAA 220 FS, Varian, Kyoto, Japan). The certified reference material Peach Leaves (NIST SRM 1547) was analyzed under the same conditions to assess the accuracy of the analytical procedure.

### 4.4. Extraction of Volatile Compounds and GC–MS Analysis

For the extraction of volatile organic compounds, 100 g of fresh samples were homogenized, mixed with 500 mL of water, and subjected to hydrodistillation using a Clevenger-type apparatus for 3 h. At the end of the distillation period, the compounds of interest were extracted from the hydrosol using 1 mL of *n*-hexane (GC grade, ≥99% purity; Sigma-Aldrich, St. Louis, MO, USA).

The volatile compounds were analyzed using a gas chromatography–mass spectrometry (GC–MS) system (GCMS-QP2020 NX, Shimadzu Corporation, Kyoto, Japan) equipped with an SH-RTx-5Sil MS fused-silica capillary column (30 m × 0.25 mm i.d.; 0.25 μm film thickness). The injector and detector temperatures were set at 260 °C. Helium was used as the carrier gas at a flow rate of 1.0 mL min^−1^. The oven temperature was programmed from 60 °C to 250 °C at a rate of 3 °C min^−1^. A 1.0 μL aliquot of the volatile fraction in *n*-hexane was injected in split mode at a split ratio of 1:10. The mass scan range was *m*/*z* 32–420, and the ionization energy was set to 70 eV [38].

Chromatographic data processing and peak detection were performed using the instrument software, LabSolutions/GCMSsolution (version 4.52, Shimadzu Corporation, Kyoto, Japan). Peak integration and baseline determination were performed using an automated peak-integration algorithm with a minimum slope threshold of 500 min^−1^. The mass spectral purity of each chromatographic peak was systematically evaluated by examining the target-ion profiles and fragmentation patterns across the peak width, confirming the absence of co-eluting compounds under the established chromatographic conditions.

Compound identification was based on two simultaneous criteria: (i) comparison of the acquired mass spectra with the NIST20 spectral library, considering similarity indices > 90%; and (ii) comparison of the experimental arithmetic retention index (AI), calculated according to Equation (2) using a series of C_6_–C_30_ *n*-alkanes (Sigma-Aldrich, St. Louis, MO, USA), with literature reference values [22,57]. Compounds showing a ΔAI < 10 units were considered consistent with the corresponding literature values. Relative abundances were calculated from the integrated peak areas in the total ion chromatogram (TIC).

(2) $$Arithmeticindex=100\times \frac{t_{c}-t_{n}}{t_{n+1}-t_{n}}+100\times n$$

where **t_c_** is the retention time of the compound of interest; **t_n_** is the retention time of the *n*-alkane eluting immediately before the compound of interest; **t_n+1_** is the retention time of the *n*-alkane eluting immediately after the compound of interest; and **n** is the number of carbon atoms in the *n*-alkane corresponding to **t_n_**.

To ensure that the identified compounds were exclusively derived from the plant matrices and to rule out process contamination, procedural blanks were analyzed in parallel. The blank procedure consisted of processing distilled water under identical hydrodistillation time and conditions, followed by hexane extraction and GC–MS analysis using the same chromatographic parameters applied to the samples.

### 4.5. Cell Viability Assay

Cell viability was assessed using the resazurin reduction assay (7-hydroxy-3H-phenoxazin-3-one 10-oxide), a simple, rapid, and low-sample-consumption method for evaluating cellular metabolic activity [34]. In this study, BEAS-2B cells (non-tumorigenic human bronchial epithelial cells; ATCC^®^ CRL-9609^TM^), obtained from the Rio de Janeiro Cell Bank (BCRJ, Duque de Caxias, RJ, Brazil; Catalog No. 0343), were used as an in vitro model for the preliminary evaluation of cellular responses and changes in cell viability. The cells were seeded in 96-well plates at a density of 1.5 × 10^4^ cells/well and incubated at 37 °C in a humidified atmosphere containing 5% CO_2_ for 24 h to allow cell adhesion. After this period, the cells were treated with samples of both species. For this purpose, the dried infructescence samples were reconstituted in DMEM/F12 medium (Gibco, Thermo Fisher Scientific, Waltham, MA, USA) to obtain a mass equivalent to that of the fresh infructescences (designated as 100% *in natura* concentration). The volume of culture medium added was equivalent to the mass of water lost during the lyophilization process, as determined from the difference between the fresh and lyophilized sample masses. Due to high density preventing 0.22 µm membrane filtration, the stock suspension was diluted in DMEM/F12 to working concentrations of 50%, 20%, 15%, and 10% of the original *in natura* infructescences equivalent prior to filtration and biological assays. The samples were then applied to the cells and incubated under the same conditions for another 24 h. For the positive control of cell death, cells were treated with 20% dimethyl sulfoxide (DMSO; Sigma-Aldrich, St. Louis, MO, USA), whereas untreated cells were used as the negative control. Following the treatment period, resazurin (Sigma-Aldrich, St. Louis, MO, USA) was added to a final concentration of 3 mM, and the plates were incubated for 3 h. Absorbance was subsequently measured at 570 and 595 nm using a Plate Chameleon microplate reader (Hidex Oy, Turku, Finland).

### 4.6. Preparation of Hydromethanolic Extracts

Approximately 2.0 g of each freeze-dried sample were first extracted with 15 mL of *n*-hexane (PA; Nuclear, São Paulo, SP, Brazil) for 30 min in an ultrasonic bath (SSBuc-6L, MyLabor, São Paulo, SP, Brazil) at 25 °C to remove nonpolar compounds. After the first extraction, the samples were filtered, and the residues were subjected to two additional extraction cycles under the same conditions. At the end of this process, the residues were allowed to evaporate to remove the remaining solvent and were subsequently extracted three times with methanol (PA; Êxodo Científica, Hortolândia, SP, Brazil) and distilled water (8:2, *v*/*v*) under the same conditions. After completion of the extraction cycles, the solvent was removed by evaporation in a fume hood under continuous exhaust at 24 °C for 24 h, yielding the dried hydromethanolic extracts of *F. benjamina* (HME-Fb) and *F. mathewsii* (HME-Fm) [38]. The extraction yield was determined gravimetrically and calculated as the ratio of the mass of the dried extract, determined after solvent removal, to the initial mass of the plant material used for extraction, multiplied by 100. Extraction blanks were prepared in parallel by subjecting the solvents to the same extraction procedures and conditions, but without the addition of plant material, and were subsequently analyzed together with the samples to monitor potential contaminants introduced during the extraction procedure. These extracts were used to determine the total phenolic and carotenoid contents and to evaluate free radical-scavenging activity.

### 4.7. Total Phenolic Content (TPC)

The TPC was determined using the Folin–Ciocalteu reagent method [58], with modifications [38]. Initially, the dry extract was solubilized in water at a concentration of 1 mg/mL. Subsequently, a 200 μL aliquot of the sample solution was added to 1.5 mL of Folin–Ciocalteu reagent (Sigma-Aldrich, St. Louis, MO, USA) diluted with water (1:10, *v*/*v*). The mixture was homogenized and kept in the dark for 5 min. Then, 1.5 mL of an aqueous sodium bicarbonate solution (60 g/L) was added, and the mixture was incubated in the dark for 90 min. After incubation, the absorbance was measured at 725 nm using an ultraviolet–visible (UV–Vis) spectrophotometer (NI 2200, Nova Instrumentos, Piracicaba, SP, Brazil). Both a solvent blank and a reagent blank were included. The solvent blank contained the corresponding solvent without reagents or sample, whereas the reagent blank contained all assay reagents but no sample solution. A calibration curve was constructed using gallic acid (Sigma-Aldrich, St. Louis, MO, USA) as the reference standard at concentrations ranging from 62.5 to 1000 μg/mL, with the equation *y* = 0.0052*x* + 0.0012 (R^2^ = 0.9999). The results were expressed as milligrams of gallic acid equivalents per gram dried extract (mg GAE/g).

### 4.8. Total Carotenoid Content (TCC)

TCC was determined by mixing the samples with water and hexane at a ratio of 1:5:6 (*v*/*v*/*v*). The mixture was vortexed for 1 min to promote carotenoid extraction and then centrifuged for 1 min to facilitate phase separation. The absorbance of the resulting supernatant was measured at 450 nm using a microplate reader (SK201, Sinothinker, Shenzhen, Guangdong, China). Hexane was used as the blank, and β-carotene (Sigma-Aldrich, ≥95% purity, St. Louis, MO, USA) was used as the reference standard for constructing the calibration curve over the concentration range of 3.9–62.5 μg/mL. The results were expressed as micrograms of β-carotene per gram of dried extract (μg/g) [59], with the equation y = 0.0253*x* + 0.0162 (R^2^ = 0.9988).

### 4.9. Radical-Scavenging Capacity (DPPH^•^ and ABTS^•+^)

Radical-scavenging capacity was evaluated using the DPPH^•^ (1,1-diphenyl-2-picrylhydrazyl; Sigma-Aldrich, St. Louis, MO, USA) radical assay [60] and the ABTS^•+^ (2,2′-azinobis(3-ethylbenzothiazoline-6-sulfonic acid); Sigma-Aldrich, St. Louis, MO, USA) radical cation assay [61], with modifications as described in [38]. For both assays, extract solutions were prepared at a concentration of 1 mg/mL in the respective solvents (Methanol or ethanol PA, Êxodo Científica, Hortolândia, SP, Brazil). For the DPPH^•^ assay, 100 μL of the methanolic extract solution was added to 3.9 mL of a methanolic DPPH^•^ solution (100 μM). The mixture was incubated in the dark for 30 min, after which the absorbance was measured at 515 nm. For the ABTS^•+^ assay, 30 μL of the sample solution was added to 3.0 mL of an ethanolic ABTS^•+^ solution (absorbance = 0.700). The mixture was incubated in the dark for 6 min, after which the absorbance was measured at 734 nm. Absorbance measurements were performed using a UV–Vis spectrophotometer (NI 2200, Nova Instrumentos, Piracicaba, SP, Brazil). Both a solvent blank and a reagent blank were included. The solvent blank contained the corresponding solvent without reagents or sample, whereas the reagent blank contained all assay reagents but no sample solution. For both assays, calibration curves were constructed using Trolox (Sigma-Aldrich, St. Louis, MO, USA) as the antioxidant standard at concentrations of 100, 500, 1000, 1500, and 2000 μM. For the DPPH^•^ assay, the calibration curve equation was *y* = −0.0006*x* + 0.9035 (R^2^ = 0.9918), whereas for the ABTS^•+^ assay, the equation was *y* = −0.0003*x* + 0.6659 (R^2^ = 0.9966). The results were expressed as micromoles of Trolox equivalents per gram of dried extract (μmol TE/g).

### 4.10. Solid-Phase Extraction (SPE)

To remove interfering substances from the hydromethanolic extracts, approximately 200.0 mg of each extract were dissolved in 5 mL of water acidified with 5% formic acid and vortex-mixed for 2 min. The resulting solutions were then filtered through 0.22 μm membrane filters. The filtered samples were loaded onto a STRATA-X solid-phase extraction (SPE) cartridge (Phenomenex; Torrance, CA, USA), which provides selectivity for aromatic compounds. Before sample loading, the cartridges were conditioned with 3 mL of MeOH (HPLC grade, Sigma-Aldrich, St. Louis, MO, USA) and equilibrated with 3 mL of distilled water. During the extraction procedure, the vacuum pump (Eco-260, Biomec, Araucária, PR, Brazil) was maintained at −5 mmHg, with a solvent flow rate of approximately one drop per second. After the entire sample solution had passed through the cartridge, 3 mL of 5% MeOH in water were applied to remove residual interfering substances. Finally, the compounds retained on the SPE cartridge were eluted with 2.0 mL of methanol. Phenolic fraction yield was determined gravimetrically and calculated as the ratio of the mass of the phenolic fraction, determined after solvent removal, to the initial mass of the dried extract subjected to fractionation, multiplied by 100. The resulting samples were designated as the phenolic fractions of *F. benjamina* (PFFb) and *F. mathewsii* (PFFm).

### 4.11. UPLC–QTOF–MS/MS Analysis

UPLC–QTOF–MS/MS data were acquired using a liquid chromatograph coupled to a quadrupole time-of-flight mass spectrometer (SYNAPT XS, Waters Corporation, Manchester, UK) equipped with an electrospray ionization (ESI) source and monitored with MassLynx software (version. 4.1, Waters Corporation, Milford, MA, USA). A 10 μL aliquot of each sample, prepared at a concentration of 1000 ppm, was injected into a Kinetex^®^ C18 core–shell column (100 mm × 2.1 mm; 1.3 μm particle size). The mobile phase consisted of water containing 0.1% ammonium hydroxide (A; Sigma-Aldrich, St. Louis, MO, USA) and methanol (B; LC-MS grade, Sigma-Aldrich, St. Louis, MO, USA), using a linear gradient from 5% to 95% B over 20 min at a flow rate of 0.20 mL min^−1^.

The mass spectrometry acquisition parameters were as follows: capillary voltage of 4 kV, source temperature of 100 °C, desolvation temperature of 400 °C, cone voltage of 30 V, cone gas flow of 50 L/h, collision energy of 10 V for MS1 and 40 V for MS2. Mass spectra were acquired in negative electrospray ionization mode (ESI−) over an *m*/*z* range of 50 to 900. Leucine-enkephalin (*m*/*z* 554.2615 [M-H]^−^) was used as the reference lock mass during acquisition. Solvent blanks and extraction blanks were analyzed under the same chromatographic and mass spectrometric conditions as the samples to monitor potential background signals and contaminants originating from the solvents, extraction procedure, or instrumental analysis. Samples were analyzed in triplicate in a randomized order.

The LC–HRMS data were processed using MassLynx software. Molecular formulas were assigned based on accurate mass measurements, allowing a mass error of up to 10 ppm. Tentative compound annotation was performed by comparing the accurate-mass data obtained from the MS and MS/MS experiments [62,63] with records available in PubChem (National Library of Medicine; https://pubchem.ncbi.nlm.nih.gov/, accessed on 10 August 2026) and the Human Metabolome Database (HMDB; https://www.hmdb.ca, accessed on 10 August 2026). Only the molecular features consistently detected across replicate analyses were included in the final list of ten annotated metabolites.

### 4.12. NMR Analysis

^1^H NMR spectra were acquired using an NMR spectrometer (Bruker Avance III HD, 11.74 T; Bruker BioSpin GmbH, Ettlingen, Germany) operating at resonance frequencies of 500.13 MHz for ^1^H and 125.8 MHz for ^13^C nuclei. For chemical profiling of the extracts, approximately 30 mg of each sample were dissolved in deuterated methanol (CD_3_OD, ≥99.8 atom % D; Sigma-Aldrich, St. Louis, MO, USA), transferred to 5 mm NMR tubes, and subjected to NMR analysis. For detailed analysis of the SPE-derived phenolic fractions, approximately 10 mg of each fraction were dissolved in 600 μL of CD_3_OD, and ^1^H NMR spectra, as well as ^1^H–^1^H COSY, ^1^H–^13^C HSQC, and ^1^H–^13^C HMBC correlation spectra, were acquired. A solvent blank consisting of CD_3_OD was also analyzed under the same NMR conditions to monitor solvent- and background-derived signals.

The spectra and two-dimensional correlation maps were processed using TopSpin software (version 4.3.0, Bruker BioSpin Inc., Billerica, MA, USA). Chemical shifts (δ) were expressed in parts per million (ppm), with tetramethylsilane (TMS; Sigma-Aldrich, St. Louis, MO, USA) and sodium 3-(trimethylsilyl)propionate (TSP-d_4_; Sigma-Aldrich, St. Louis, MO, USA) referenced to 0.00 ppm as chemical shift references and coupling constants (J) were reported in hertz (Hz). The assignment of characteristic signals was supported by comparison with literature data [23] and entries in the Human Metabolome Database (HMDB, https://www.hmdb.ca, accessed on 10 August 2026) [64].

### 4.13. Statistical Analysis

All statistical analyses were performed using JASP software (version 0.96, University of Amsterdam, Amsterdam, The Netherlands). Quantitative experimental results are expressed as mean ± standard deviation (SD) of three independent biological replicates. Each biological replicate consisted of an independently processed sample obtained from a separate biological sampling unit. Before parametric analyses, the assumptions of normality and homogeneity of variances were assessed using the Shapiro–Wilk and Levene’s tests, respectively. Given the small sample size (n = 3), these tests were interpreted cautiously, and a non-significant result was considered as providing no statistically significant evidence of a violation of the corresponding assumption (*p* > 0.05).

For analyses involving comparisons between two groups, including proximate composition, mineral composition, total phenolic content, and free-radical-scavenging capacity, Student’s *t*-test was applied (*p* < 0.05). For the cell viability assay, the effects of the *Ficus* samples were evaluated using one-way analysis of variance (ANOVA), considering each species × concentration combination as a separate group, followed by Tukey’s HSD test for multiple comparisons (*p* < 0.05). The control and DMSO groups were also included in the analysis.

## 5. Conclusions

This study provides the first integrated characterization of the nutritional, mineral, volatile, and phenolic profiles, as well as the free-radical-scavenging capacity and effects on cell viability, of the infructescences of *F. benjamina* and *F. mathewsii*. The results demonstrated that these poorly investigated species exhibit distinct and complementary chemical profiles. *F. benjamina* showed higher carbohydrate and energy contents, as well as higher concentrations of Mg, Ca, Mn, Zn, and Fe, whereas *F. mathewsii* had a higher moisture content and was characterized by a higher total phenolic content and greater free-radical-scavenging capacity. Its total phenolic content and DPPH^•^ radical-scavenging capacity were approximately 4.3- and 3.3-fold higher, respectively, than those of *F. benjamina.* The BEAS-2B cell assay showed no significant reduction in cell viability under most of the tested conditions, although *F. benjamina* reduced cell viability to approximately 50% at the highest concentration tested. Therefore, these findings provide only preliminary information on cellular responses and should not be interpreted as evidence of safety. Likewise, the results of the DPPH^•^ and ABTS^•+^ assays reflect in vitro free-radical-scavenging capacity and do not establish in vivo antioxidant efficacy.

The volatile fractions of both species showed a high relative representation of sesquiterpenes, although differences were observed in the relative distribution of the detected compounds. *F. benjamina* showed larger relative peak areas for *β*-selinene, *δ*-cadinene, and *cis*-*α*-bergamotene, while *β*-elemene, *β*-farnesene, and *α*-selinene showed larger relative peak areas in *F. mathewsii*. UPLC–QTOF–MS/MS analysis allowed the identification of ten phenolic metabolites, including flavonols, flavones, a flavanone, and a phenolic acid. Complementary NMR analyses provided additional structural evidence for kaempferol and chlorogenic acid in the phenolic fractions.

Overall, the findings meet the objectives of this study and demonstrate that the two species exhibit distinct chemical profiles. *F. benjamina* was distinguished by its higher mineral content, whereas *F. mathewsii* exhibited higher phenolic content and free-radical-scavenging capacity. This study expands the still limited knowledge of these frequently discarded infructescences and contributes to their chemical and biological characterization. However, further studies addressing nutrient and phytochemical bioavailability, individual metabolite quantification, comprehensive toxicological evaluation, seasonal and geographic variability, and the underlying biological mechanisms are needed before their potential food, nutraceutical, or pharmaceutical applications can be adequately assessed.

## Figures and Tables

**Table 1:** Proximate composition (g/100 g) and energy value (kcal/100 g) of fresh *F. benjamina* and *F. mathewsii* infructescences.

Sample	Moisture	Carbohydrates	Protein	Ash	Lipids	Energy
*F. benjamina*	66.30 ± 1.08 ^b^	30.39 ± 1.04 ^a^	1.08 ± 0.04 ^a^	1.56 ± 0.05 ^a^	0.67 ± 0.03 ^a^	131.93 ± 4.40 ^a^
*F. mathewsii*	76.09 ± 0.99 ^a^	21.51 ± 0.93 ^b^	0.94 ± 0.04 ^b^	0.99 ± 0.02 ^b^	0.47 ± 0.02 ^b^	94.00 ± 3.91 ^b^

Values are expressed as mean ± standard deviation (n = 3 independent biological replicates). Different superscript letters within the same column indicate significant differences among means according to Tukey’s test (*p* < 0.05).

**Table 2:** Mineral composition of freeze-dried *F. benjamina* and *F. mathewsii* infructescences.

Sample	Mg	K	Ca	Zn	Mn	Fe	P	Cu
	g kg^−1^			mg kg^−1^				
*F. benjamina*	14.34 ± 0.18 ^a^	12.23 ± 0.22 ^a^	9.86 ± 0.10 ^a^	95.50 ± 1.50 ^a^	42.00 ± 2.00 ^a^	23.50 ± 0.50 ^a^	Tr.	Tr.
*F. mathewsii*	12.05 ± 0.03 ^b^	11.39 ± 0.09 ^a^	6.86 ± 0.01 ^b^	82.00 ± 2.00 ^b^	11.50 ± 1.50 ^b^	14.50 ± 1.50 ^b^	Tr.	Tr.

Values are expressed as mean ± standard deviation (n = 3 independent biological replicates). Different superscript letters within the same column indicate significant differences among means according to Tukey’s test (*p* < 0.05). Tr.: trace.

**Table 3:** Relative peak area (%) of volatile compounds detected in *Ficus benjamina* (Fb) and *Ficus mathewsii* (Fm) infructescences.

No.	RT	MF	Compound	Relative Peak Area (%)		AI		SI
				Fb	Fm	Exp.	Ref.	
*Sesquiterpenes*				52.80 ± 1.12	47.66 ± 0.11			
1	20.79	C_15_H_24_	*δ*-elemene	2.46 ± 0.11	–	1333	1335	96
2	22.35	C_15_H_24_	*α*-copaene	–	2.86 ± 0.07	1370	1374	94
3	23.05	C_15_H_24_	*β*-elemene	3.43 ± 0.08	7.56 ± 0.32	1386	1390	92
4	23.94	C_15_H_24_	*cis*-*α*-bergamotene	5.42 ± 0.15	1.59 ± 0.03	1407	1411	92
5	24.27	C_15_H_24_	*C*aryophyllene	2.08 ± 0.06	3.07 ± 0.05	1415	1417 (*E*) or 1408 (*Z*)	95
6	24.91	C_15_H_24_	*trans*-*α*-bergamotene	3.45 ± 0.14	–	1431	1432	95
7	25.67	C_15_H_24_	*β*-Farnesene	4.26 ± 0.12	5.83 ± 0.26	1449	1454 (*Z*) or 1440 (*E*)	98
8	26.79	C_15_H_24_	Germacrene D	–	2.14 ± 0.06	1477	1480	94
9	27.17	C_15_H_24_	*β*-selinene	7.34 ± 0.33	4.59 ± 0.13	1486	1489	95
10	27.23	C_15_H_24_	Viridiflorene	2.41 ± 0.09	2.71 ± 0.04	1487	1496	96
11	27.33	C_15_H_24_	*α*-selinene	3.05 ± 0.07	5.83 ± 0.13	1490	1498	94
12	27.49	C_15_H_24_	*α*-muurolene	3.36 ± 0.15	2.21 ± 0.04	1494	1500	95
13	27.92	C_15_H_24_	*β*-bisabolene	3.67 ± 0.08	–	1504	1505	95
14	28.09	C_15_H_24_	*γ*-cadinene	2.22 ± 0.07	4.72 ± 0.14	1508	1513	96
15	28.36	C_15_H_24_	*δ*-cadinene	5.92 ± 0.20	2.86 ± 0.09	1515	1522	92
16	28.71	C_15_H_24_	*γ*-bisabolene	3.72 ± 0.09	1.69 ± 0.10	1524	1529 (*E*) or 1514 (*Z*)	92
*Sesquiterpenoids*				12.78 ± 0.28	17.65 ± 0.28			
17	32.57	C_15_H_26_O	*γ*-eudesmol	2.05 ± 0.09	4.87 ± 0.10	1623	1630	92
18	33.43	C_15_H_26_O	*α*-eudesmol	3.05 ± 0.07	3.74 ± 0.12	1646	1652	92
19	33.59	C_15_H_26_O	Neointermedeol	1.67 ± 0.30	5.59 ± 0.14	1650	1658	96
20	35.06	C_15_H_24_O	Isovalencenol	–	3.45 ± 0.09	1790	1793	95
*Esters*				5.04 ± 0.07	10.91 ± 0.03			
21	43.13	C_17_H_34_O_2_	Methyl palmitate	–	3.05 ± 0.11	1921	1921	92
22	48.45	C_19_H_34_O_2_	Methyl linoleate	1.67 ± 0.10	3.16 ± 0.08	2089	2095	96
23	50.49	C_20_H_36_O_2_	Ethyl Linoleate	3.38 ± 0.11	4.71 ± 0.12	2157	2159	94

Values are expressed as mean ± standard deviation (n = 3 independent biological replicates). RT: Retention time in minutes. MF: Molecular Formula. AI: Arithmetic Index. SI: Similarity Index. Exp.: Experimental Value. Ref.: Reference [22]. Compounds presenting two theoretical arithmetic indices in the second-to-last column indicate the presence of two isomers (*E*/*Z*) with high similarity in fragmentation patterns and arithmetic indices, which could not be differentiated due to the methodological limitations of this study.

**Table 4:** Radical-scavenging capacity (DPPH^•^ and ABTS^•+^), and total phenolic content of hydromethanolic extracts from *Ficus benjamina* and *Ficus mathewsii* infructescences.

Sample	DPPH^•^ (μmol TE/g)	ABTS^•+^ (μmol TE/g)	TPC (mg GAE/g)
*F. benjamina*	300.5 ± 2.1 ^b^	1152.4 ± 18.9 ^b^	152.7 ± 4.8 ^b^
*F. mathewsii*	998.3 ± 4.6 ^a^	1572.9 ± 21.0 ^a^	659.1 ± 4.8 ^a^

Values are expressed as mean ± standard deviation (n = 3 independent biological replicates). Different superscript letters within the same column indicate significant differences among means according to Student’s *t*-test (*p* < 0.05). TPC: Total phenolic content; TCC: total carotenoid content; mg GAE/g: milligrams of gallic acid equivalents per gram of sample; μmol TE/g: micromoles of Trolox equivalents per gram of sample.

## Data Availability

The original contributions presented in this study are included in the article. Further inquiries can be directed to the corresponding authors.
